# Exploring the Sheep *MAST4* Gene Variants and Their Associations with Litter Size

**DOI:** 10.3390/ani14040591

**Published:** 2024-02-11

**Authors:** Nazar Akhmet, Leijing Zhu, Jiajun Song, Zhanerke Akhatayeva, Qingfeng Zhang, Peng Su, Ran Li, Chuanying Pan, Xianyong Lan

**Affiliations:** 1 Key Laboratory of Animal Genetics, Breeding and Reproduction of Shaanxi Province, College of Animal Science and Technology, Northwest A&F University, Yangling 712100, China; akhmetnazar@163.com (N.A.); zhuleijingjingshu@163.com (L.Z.); ran.li1986@hotmail.com (R.L.); panyu1980@126.com (C.P.); 2 Scientific Research Institute of Sheep Breeding Branch, Kazakh Scientific Research Institute of Animal Husbandry and Fodder Production, Mynbaev 040622, Kazakhstan; akhatayevazhanerke@163.com; 3 Tianjin Aoqun Sheep Industry Academy Company, Tianjin 300000, China; zhangqf@aoqunmuye.com; 4 National Germplasm Center of Domestic Animal Resources, Institute of Animal Science, Chinese Academy of Agricultural Sciences (CAAS), Beijing 100193, China; 82101231269@caas.cn

**Keywords:** sheep, the *MAST4* gene, insertion/deletion, litter size

## Abstract

**Simple Summary:**

Increasing the fertility of sheep remains one of the crucial issues of modern sheep breeding. Recently, the microtubule associated serine/threonine kinase family member 4 (*MAST4*) gene has been proposed to have an impact on sheep prolificacy traits. Moreover, the *MAST4* gene is known for its role in both male and female reproduction. However, it has not been previously linked to sheep fertility traits. Therefore, the focus of our research was to determine whether this gene affects litter size traits in sheep. To address this problem, whole-genome sequencing (WGS) methodology (*n* = 1507) in 26 different sheep breeds worldwide and polymerase chain reaction (PCR) genotyping of the *MAST4* gene polymorphisms were conducted in (*n* = 566) Australian white (AUW) sheep. The findings indicated that the detected polymorphisms within this gene were presented in all 26 breeds, except for P5-del-24 bp, which was presented in 24 out of 26 breeds. Notably, all InDel loci were found to be associated with sheep traits (*p* < 0.05) in AUW sheep, making them effective molecular marker loci for sheep breeding.

**Abstract:**

The economic efficiency of sheep breeding can be improved by enhancing sheep productivity. A recent genome-wide association study (GWAS) unveiled the potential impact of the *MAST4* gene on prolificacy traits in Australian White sheep (AUW)). Herein, whole-genome sequencing (WGS) data from 26 different sheep breeds worldwide (*n* = 1507), including diverse meat, wool, milk, or dual-purpose sheep breed types from China, Europe, and Africa, were used. Moreover, polymerase chain reaction (PCR) genotyping of the *MAST4* gene polymorphisms in (*n* = 566) Australian white sheep (AUW) was performed. The 3 identified polymorphisms were not homogeneously distributed across the 26 examined sheep breeds. Findings revealed prevalent polymorphisms (P3-ins-29 bp and P6-del-21 bp) with varying frequencies (0.02 to 0.97) across 26 breeds, while P5-del-24 bp was presented in 24 out of 26 breeds. Interestingly, the frequency of the P3-ins-29 bp variant was markedly higher in Chinese meat or dual-purpose sheep breeds, while the other two variants also showed moderate frequencies in meat breeds. Notably, association analysis indicated that all InDels were associated with AUW sheep litter size (*p* < 0.05). These results suggest that these InDels within the *MAST4* gene could be useful in marker-assisted selection in sheep breeding.

## 1. Introduction

Sheep breeding is an important branch of livestock production. Sheep provide valuable raw materials such as sheepskin and wool for the textile industry, in addition to being a source of mutton, fat, and milk—commodities that enjoy high demand among the global population [1]. Over the course of more than 10,000 years, humans have engaged in the breeding of sheep [2]. Presently, the worldwide sheep population stands at 1173 million, with Asia contributing 512 million heads, accounting for 43.6% of the global count [3]. Given the fact that the world population is rapidly growing, as a consequence, the need for cost-effective food products has increased significantly [4,5]. Sheep breeding in Asia becomes pivotal in meeting these social demands, contributing to stable and secure food supplies. It also stabilizes food supplies and brings economic opportunities, impacting local communities and fostering growth. Moreover, reproduction is an integral component of sheep production and plays a crucial role in the sheep industry [6,7]. Therefore, identifying the various factors affecting these processes is of paramount importance. Interestingly, with the advancement of genetics and the widespread of marker-assisted selection in animal breeding it has become possible to identify specific candidate genes responsible for carrying out various processes in animal organisms [8].

Marker-assisted selection (MAS) represents a highly lucrative and remarkably efficient approach to animal breeding, thereby conferring notable benefits, including enhanced economic and temporal efficiency [9]. This method has emerged as a pivotal instrument within the field of animal genetics, instigating a transformative shift in conventional breeding practices. Leveraging the potential of molecular markers and genomic information, molecular breeding facilitates the identification and precise selection of desirable traits with a high level of accuracy and precision [10].

Recently, a genome-wide association study (GWAS) conducted by our team has revealed that the microtubule-associated serine/threonine kinase family member 4 (*MAST4*) gene probably has a significant effect on the litter size traits of AUW sheep. AUW sheep is a coarse wool specialized mutton sheep breed and is now distributed in Australia, China, and other places [11]. The *MAST4* gene belongs to the *MAST* kinase family, which is characterized by their association with microtubules and their involvement in regulating microtubule dynamics. Microtubules are structures within cells that play a role in cell shape, intracellular transport, and cell division [12,13]. Additionally, the initial documentation of the *MAST4* gene was presented in the research conducted by Sun and colleagues [14]. Of great interest, functional investigations have subsequently illustrated that *MAST4* plays a significant role in the pathological mechanisms concerning the central nervous system [15,16]. Moreover, recent research has indicated that the regulation of the *MAST4* gene plays a vital role in maintaining spermatogonial stem cells involved in spermatogenesis, which encompasses the intricate progression of male germ cell development culminating in the intricate differentiation and maturation processes necessary for the production of functional spermatozoa [17]. Furthermore, it is noteworthy that the study conducted by Cui et al. shows that *MAST4* demonstrates responsiveness to estrogen stimulation [18]. Nonetheless, the specific contribution of the *MAST4* gene to the litter size of domestic animals remains unexplored. As previously noted, the *MAST4* gene has been implicated in its association with the litter size of AUW sheep. In light of these considerations, we have postulated that the *MAST4* gene may exert an influence on the litter size of sheep. Therefore, the primary objective of our study is to delve into the potential impact of the *MAST4* gene on the litter size of AUW sheep and investigate the frequencies of this gene variant across different sheep breeds. By undertaking a comprehensive investigation, this study aims to fill a current gap in our understanding of the *MAST4* gene’s contribution to sheep prolificacy.

The significance of this research lies in its potential to improve sheep breeding practices. Understanding the genetic intricacies, particularly the role of the *MAST4* gene, can pave the way for more efficient and productive breeding. The working hypothesis of our research is InDel variations at a given gene are significantly associated with litter size in AUW sheep.

## 2. Materials and Methods

### 2.1. Ethics Statement

This study was conducted in strict accordance with the Regulations for the Administration of Affairs Concerning Experimental Animals (Ministry of Science and Technology, China, 2004). All experimental procedures were performed in accordance with the guidelines of the Faculty Animal Policy and Welfare Committee of Northwest A&F University (protocol No. NWAFU-314020038) for the use and care of animals in research. Sample collection was conducted in accordance with China’s national standards: the Guidelines on Welfare and Ethical Review for Laboratory Animals (GB/T35892-2018) [19].

### 2.2. Animals and Data

**Figure 1:**
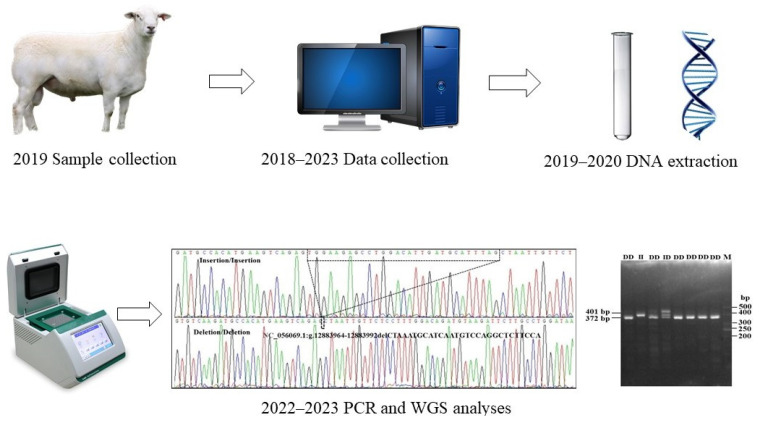
Experimental protocol.

In the present study, the focus was on examining the polymorphisms within the *MAST4* gene and assessing the genetic diversity of these polymorphisms. To ensure a representative sample, a randomly selected cohort of 566 AUW sheep was involved. Data on litter size were available for 394 out of the total 566 AUW ewes. These data were further classified according to parity, allowing for a more comprehensive analysis. Moreover, we employed Whole Genome Sequencing (WGS) data encompassing 1507 sample sets of different sheep breeds across the world. Sample collection information related to sheep populations used to evaluate the frequencies of the *MAST4* variants in different sheep breeds worldwide was previously described by Li et al. (2023) [20]. In addition, the detailed information regarding experiment design is illustrated in the Figure 1.

### 2.3. Genomic DNA Extraction

Genomic DNA (gDNA) was extracted from ear tissues and whole blood samples using a phenol–chloroform method, which has been widely employed for this purpose [21]. The extracted DNA underwent rigorous assessment for both concentration and purity using a NanoDrop 2000 spectrophotometer (Thermo Scientific, Waltham, MA, USA), a standard instrument for DNA analysis. Subsequently, to ensure consistency across samples, each DNA sample was appropriately diluted to achieve a standardized concentration of 20 ng/μL, following established protocols. Finally, for long-term preservation, the samples were carefully stored at a temperature of −40 °C, which is commonly employed for maintaining DNA stability.

### 2.4. Primer Design and PCR-Based Genotyping

The primer sequences were designed for the ovine *MAST4* gene using the reference sequence NC_056069.1 from NCBI. Information about predicted insertion/deletions was derived from the Ensembl databases through the utilization of the NCBI Primer Blast software (https://www.ncbi.nlm.nih.gov/tools/primer-blast/index.cgi?LINK_LOC=BlastHome, accessed on 6 June 2023). In order to identify novel indel loci within the sheep *MAST4* gene, a total of nine primer pairs were designed and synthesized by the Sangon Biotech Company (Shanghai, China) (Table 1). To facilitate the detection of these novel InDel loci, a DNA pool was constructed using a random selection of 50 individuals. For the Touch-down PCR program, a reaction volume of 13 μL was employed. The PCR amplification protocol consisted of an initial denaturation step at 95 °C for 5 min, followed by 18 cycles of denaturation at 94 °C for 30 s, annealing at 68 °C for 30 s, and extension at a rate of 1000 bp/min at 72 °C. This was followed by an additional 30 cycles of denaturation at 94 °C for 30 s, annealing at 50 °C for 30 s, and extension at 72 °C for 20 s, with a final extension step at 72 °C for 10 min. The reaction mixture was then cooled to 4 °C. The PCR products were subsequently separated on a 3.5% agarose gel and subjected to sequencing analysis by the Sangon Company (Shanghai, China).

### 2.5. Whole-Genome Sequences (WGS) Data and Bioinformatic Analysis

To investigate the distribution of polymorphisms within the *MAST4* gene across diverse global sheep breeds, sequencing data sets for 1507 individuals representing 26 breeds from different geographic regions (China, Europe, and Africa) were acquired through the implementation of whole-genome sequencing (WGS) methodology. To access genetic frequencies, we collected 957 sheep samples from China, 173 from Africa, 344 from Europe, and 33 wild sheep based on the sample size of breeds, which was more than 30 (n ≥ 30). Comprehensive details pertaining to the genotyping procedures can be found in the research conducted by Li et al. (2023) [19]. 

### 2.6. Statistical Analyses

The genotypic frequencies, allelic frequencies, Hardy–Weinberg equilibrium (HWE), linkage disequilibrium (LD), and genetic parameters of the identified indel loci were analyzed using the SHEsis platform available at http://analysis.bio-x.cn/myAnalysis.php (accessed on 6 June 2023). The association between the polymorphic site and litter size was evaluated using a general linear model: Y*_ijk_* = μ + P*_i_* + G*_j_* + e*_ijk_*. In this model, Y*_ijk_* represents the phenotypic value of litter size, μ denotes the overall population mean, P*_i_* represents the fixed effect of parity, G*_j_* represents the fixed effect of genotype, and e*_ijk_* represents the random error. The association analysis was conducted using either the independent t-test or one-way ANOVA analysis through SPSS software (version 25.0, IBM Corporation, New York, NY, USA). The genotypic distribution among single- and multi-lamb ewes within the Australian White (AUW) sheep population was assessed through a Chi-square (χ2) test [22]. 

## 3. Results

### 3.1. Indel Genotyping and Sequencing

**Figure 2:**
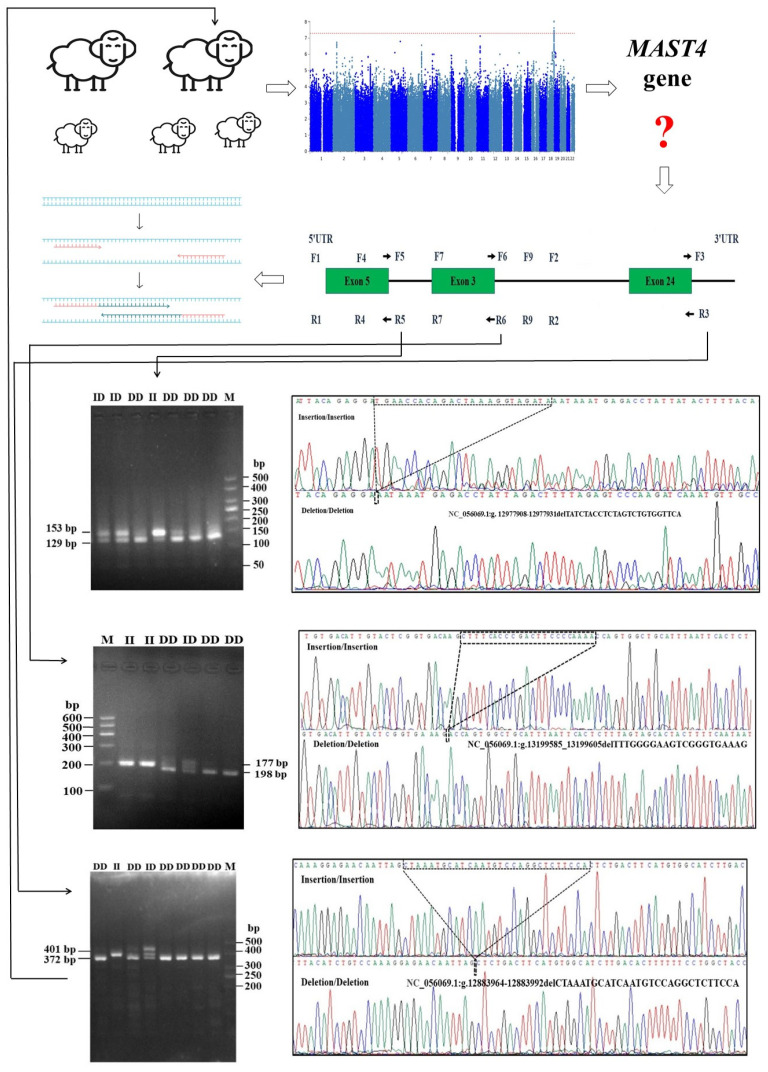
The research design.

Based on the information derived from Ensembl and NCBI databases, a total of nine pairs of primers were designed. After conducting DNA analysis, three indel mutations in AUW sheep, namely, P3-ins-29 bp, P5-del-24 bp, and P6-del-21 bp (Figure 2), were identified within the *MAST4* gene. These mutations exhibited three different genotypes: insertion (II), insertion/deletion (ID), and deletion/deletion (DD). 

The identification of the *MAST4* gene was achieved through a comprehensive genome-wide association study (GWAS). Subsequently, we conducted an exhaustive search across the Ensembl and NCBI databases to explore potential genetic loci and designed nine primer pares. This comprehensive approach led to the discovery of the P3-ins-29 bp, P5-del-24 bp, and MAST4-P6-del-21 bp indel variants. Furthermore, this was followed by PCR analysis to confirm our findings. Agarose gel electrophoresis (3.5%) of allele-specific polymerase chain reaction (PCR) product and sequencing map of the MAST4-P3-ins-29 bp, MAST4-P5-del-24 bp, and MAST4-P6-del-21 bp indel variants within the *MAST4* gene in AUW sheep. II: homozygous insertion genotype; DD: homozygous deletion genotype; and ID: heterozygous insertion/deletion genotype. The sequence with the black border is a different sequence. The top band in the electrophoresis pattern of ID represents a heteroduplex.

### 3.2. Genotypic c and Allelic Frequencies

The diversity in genetics for the indel polymorphism locus (as shown in Table 2) was examined. The findings revealed that in AUW sheep, the frequency of the ID genotype (0.674) was higher compared to the II and DD genotypes. Specifically, the “D” allele had a higher frequency than “I” in P3-del-29 bp and P5-ins-24 bp variants. In contrast, for P6-del-21 bp, the “D” allele was less frequent than “I”. The observed genotype distribution for the variants P3-del-29 bp and P5-del-24 bp did not fit with the Hardy–Weinberg equilibrium at a significance level of *p* < 0.05. Conversely, the variant P6-del-21 bp confirmed the Hardy–Weinberg equilibrium (*p* > 0.05). By assessing the PIC value, it was determined that all indels displayed a high level of variability in the AUW sheep population (PIC > 0.25).

In addition, this research outlines the genetic variations in the *MAST4* gene among different sheep breeds from various regions around the world. Genotyping results further unveil that the polymorphisms are present in all 26 breeds, except for P5-del-24 bp, which exhibits segregation in 24 out of the 26 breeds, demonstrating varied frequencies ranging from 0.02 to 0.97 (Table 3). Interestingly, the distribution of allele frequencies for the P3-del-29 bp variant within the *MAST4* gene indicates that Tan and Yunnan sheep manifest the highest frequencies, surpassing other breeds across diverse regions.

### 3.3. Linkage Disequilibrium

**Figure 3:**
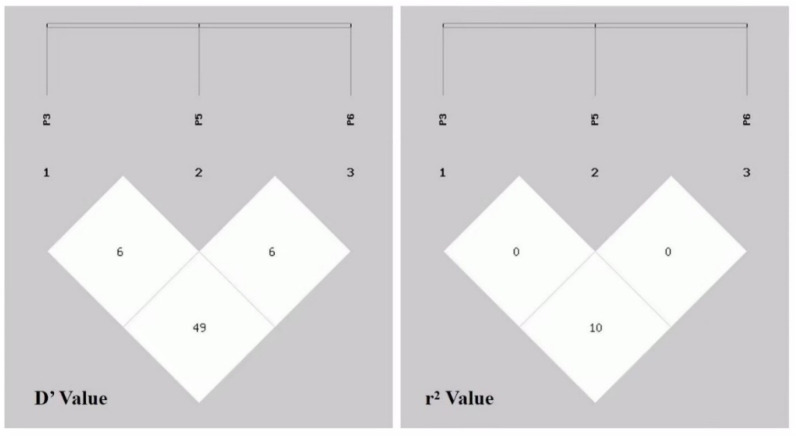
Linkage disequilibrium analysis of variants within the *MAST4* gene in AUW sheep. P3, P5, P6 mean: P3-ins-29 bp, P5-del-24 bp, and P6-del-21 bp loci.

Considering the fact that all three mutations were situated within the same gene, we speculated about the potential linkage among them. In order to investigate the potential linkage between the variant loci of *MAST4*, we conducted a linkage disequilibrium (LD) analysis using the SHEsis online platform. The LD analysis revealed that the D′ and r^2^ values for the association between the P3-ins-29 bp, P5-del-24 bp, and P6-del-21 bp loci within the *MAST4* gene were 0.061, 0.491, 0.069 and 0.003, 0.106, 0.001, respectively, suggesting that these three mutations were not significantly correlated (Figure 3). 

### 3.4. Association of the MAST4 Gene with Litter Size in AUW Sheep

Remarkably, the association analysis revealed compelling results regarding the P3-ins-29 bp site of the *MAST4* gene. Specifically, individuals with the DD genotype displayed a higher second parity litter size compared to individuals with the II and ID genotypes (*p* < 0.05; Table 4). Thus, the DD genotype was identified as the favorable genotype for the P3-ins-29 bp site. Additionally, regarding the P5-del-24 bp site of the *MAST4* gene, individuals with the II genotype exhibited a significantly higher first-parity litter size compared to individuals with the ID and DD genotypes (*p* < 0.05; Table 4). Therefore, for the P5-del-24 bp site in *MAST4*, the II genotype was recognized as the favorable genotype. Concerning the P6-del-21 bp site within the *MAST4* gene, significant connections were found with both the average litter size and the total number of lambs. Notably, the DD genotype was the superior genotype (*p* < 0.05; Table 4). In addition, the information on genotypic distribution between mothers of single-lamb and multi-lamb is given in the Table 5.

## 4. Discussion

In our recent GWAS study, we discovered compelling evidence pointing to the significant influence of the *MAST4* gene on prolificacy traits in AUW sheep. This finding prompted our hypothesis about a significant effect of InDel genotypes of the *MAST4* gene on different parity litter sizes in AUW ewes. The exploration of variations within the *MAST4* gene and their potential links to litter size in the examined population revealed a significant gap in existing studies. Notably, this study stands out as the first to report a substantial association between indel polymorphisms in the *MAST4* gene and litter size in sheep. 

Given the escalating global population and the need for cost-effective means to sustain, marker-assisted selection (MAS) is gaining attention as a promising approach [23]. MAS offers several advantages over traditional breeding methods, primarily in terms of time efficiency. This integrated approach effectively bridges the gap between genotype and phenotype, enabling the identification of valuable genetic resources for targeted utilization in practical breeding programs [24]. Hence, in alignment with the advancements in MAS and recognizing its potential to revolutionize breeding methodologies, we have employed MAS in our study, embracing its capabilities to enhance precision and efficacy in our research.

To validate our hypothesis, we conducted PCR genotyping in AUW sheep and found three polymorphisms. Of interest, population genetic diversity characteristics play a pivotal role in assessing the genetic potential and conservation of breeds with distinctive traits [25]. Further, we evaluated the distributions of these polymorphisms across different sheep breeds globally using whole-genome sequencing (WGS) data, strengthening our research and supporting the theory of these polymorphisms affecting sheep litter size traits. Moreover, to enhance the significance of our results in evaluating the *MAST4* gene variant frequencies, we have chosen breeds with a sample size of no less than 30 individuals from diverse regions worldwide. Additionally, our study included various types of sheep breeds, including wool, meat, milk, and dual purpose. Intriguingly, the frequencies of the P3-ins-29 bp variant were predominantly higher in Chinese meat-producing or dual-purpose breeds. This strengthens our research, especially considering the GWAS study, which revealed that the *MAST4* gene specifically affects litter size in AUW sheep, with frequencies of meat-purpose breeds being higher than others.

Moreover, the deviation from HWE is unlikely to be attributed to genetic drift or migration, suggesting the presence of other influencing factors on the genotype distribution [26]. Furthermore, despite all three mutations being situated within the same gene, *MAST4*, we did not find any linkage disequilibrium (LD) between them. Their close proximity in the gene did not result in significant LD among the mutations. 

On top of that, the significant effects of these polymorphisms can be explained by functions of the *MAST4* gene. Spermatogonial stem cells (SSCs) are a type of stem cell located in the testes, the male reproductive organs. These cells give rise to spermatocytes through a process of meiotic division. Upon maturation, spermatocytes develop into spermatozoa, which are essential for male fertility and the process of reproduction. SSCs play a crucial role in maintaining the continual production of sperm throughout a male’s life [27,28]. Interestingly, *MAST4* is intricately linked to the maintenance of Sertoli cells, the exclusive somatic cell type found in the tubules. It interacts directly with Spermatogonial Stem Cells (SSCs) and exerts control over their proliferation and differentiation through secreted factors, including glial cell lineage neurotrophic factor and the E26 transformation-specific (ETS) variant 5 transcription factor, also known as (ERM). *MAST4* also plays a role in regulating the cell cycle of SSCs by phosphorylating ERM in Sertoli cells, thereby influencing the transcription of Cxcl12, an ERM target gene [17]. Thus, the positive effects of the *MAST4* gene on litter size could be explained by the functional importance of this gene in the maintenance of Sertoli cells.

Moreover, new research has shown that the *MAST4* gene is linked to how much fat Nelore cattle store [29]. In light of this discovery, it makes sense to call the *MAST4* gene a “pleiotropic gene” since it affects both fat levels and litter size. Even though these effects show up in different groups of animals, we still call it pleiotropic. Pleiotropy is a common genetic idea where one gene affects lots of traits that might seem different. The fact that the *MAST4* gene affects fat storage and offspring count in different animals shows that it is a pleiotropic gene.

In addition to this, all mutations were situated in an untranslated region. However, we postulate that mutations occurring within the untranslated regions of the current gene could also impact the observed phenotype. This is because introns play a crucial role in the molecular mechanisms of genetic information implementation [30]. It is now understood that introns contain sequences essential for gene function and encompass various regulatory elements that influence gene expression. These elements include transcription binding factors, alternative promoters, and enhancers [31,32]. Remarkably, our results indicated a significant association between these three mutations and the litter size of sheep, making them viable candidates for MAS in future breeding programs.

In summary, this investigation marks the inaugural exploration into the impact of indel polymorphisms within the *MAST4* gene on sheep litter size. Our findings, revealing potential associations between these polymorphisms and litter size, offer valuable insights for the formulation of future sheep breeding strategies, particularly emphasizing MAS as a prospective avenue for enhancing breeding practices. Moreover, variants within the *MAST4* gene were distributed in all 26 investigated breeds, except for the P5-del-24 bp variant, which was present in 24 breeds. Furthermore, the P3-ins-29 bp variant exhibited significantly higher frequencies in Chinese meat or dual-purpose sheep breeds. Meanwhile, the other two variants displayed moderate frequencies, also in meat breeds. 

## 5. Conclusions

In conclusion, the extensive examination of *MAST4* gene variants across 26 diverse breeds underscores their widespread distribution. Particularly noteworthy is the significantly higher frequency of the P3-ins-29 bp variant in Chinese meat or dual-purpose sheep breeds. Moreover, the polymorphisms within the *MAST4* gene in AUW ewes were significantly associated with litter size. Recognizing the importance of the *MAST4* gene, these findings provide a foundation for future research, exploring the underlying causal mutation and advocating for the application of MAS in sheep breeding practices.

## Figures and Tables

**Table 1:** PCR primer sequences of the *MAST4* gene for amplification.

Loci	Primer Sequences (5′–3′)	Tm (°C)	Product Sizes (bp)
P1-del-35 bp	F: TATCCTTAATCAGCCCCACGG	59.3	161/126
	R: TCCACTGTTCACCCAGATCAA	58.9	
P2-del-31 bp	F: AGCTGAGGCTGATGTTGGAG	59.7	355/324
	R: CGGACAGCACTGACATTGAC	59.2	
**P3-ins-29 bp**	**F: AGGACAAGAGTCAGGGGCAT**	**60.5**	**401/372**
	**R: GGGTGCAGTGAATAACACGG**	**59.2**	
P4-del-29 bp	F: CTCCCGACTTGAAACAATCTGC	59.8	299/270
	R: CCTAACCAAGGGTGGACAGAG	59.7	
**P5-del-24 bp**	**F: TGCCTACCACAGTATCACTTCAA**	**59.4**	**153/129**
	**R: ATGGGGCAACATTTGATCTTGG**	**59.5**	
**P6-del-21-bp**	**F: GACCATCCTGTCCCCTTTAGAC**	**59.8**	**198/177**
	**R: TTCCAAAGGGTATCTTTATCCTGC**	**58.5**	
P7-del-21 bp	F: GACCATCCTGTCCCCTTTAGAC	59.8	198/177
	R: TTCCAAAGGGTATCTTTATCCTGC	58.5	
P8-del-24 bp	F: TTAAGTGAAGCCTGGTACCTTCA	59.3	424/403
	R: ACCACTCACCTTCCAGAGGAT	60.2	
P9-del-21 bp	F: GCAGAGGTCAGGAAAGCAGA	59.6	141/120
	R: TCTCCATTCTGGGTGCTGTG	59.6	

Note: F: forward; R: reverse; P: primer. Bold means polymorphisms.

**Table 2:** Genetic diversity parameters for *MAST4* polymorphisms in AUW sheep.

Loci	Sample Sizes	Genotypic Frequencies			Allelic Frequencies		HWE	Molecular Genetic Parameters			
		II	ID	DD	I	D	*p* Values	Ho	He	Ne	PIC
P3-del-29 bp	566	0.159	0.611	0.229	0.464	0.535	*p* < 0.05	0.502	0.497	1.990	0.373
P5-ins-24 bp	378	0.063	0.674	0.261	0.400	0.599	*p* < 0.05	0.519	0.480	1.924	0.365
P6-del-21 bp	329	0.580	0.358	0.060	0.759	0.240	*p* > 0.05	0.635	0.364	1.574	0.298

Note: Ho, homozygosity; He, heterozygosity; Ne, effective allele numbers; PIC, polymorphism information content; HWE, Hardy–Weinberg equilibrium; II, insertion/insertion; ID, insertion/deletion; DD, deletion/deletion.

**Table 3:** The frequencies of the *MAST4* variants in different sheep breeds worldwide.

Species/Regions	Sample Size	Breeds	Breed Type	Frequencies of P3-ins-29 bp	Frequencies of P5-del-24 bp	Frequencies of P6-del-21 bp
Wild	n = 33	Mouflon	Wild sheep	0.95	0.14	0.12
China	n = 129	Hu	Meat	0.94	0.15	0.24
	n = 41	Altay	Meat	0.85	0.12	0.26
	n = 33	Bashibai	Wool–meat	0.81	0.20	0.29
	n = 31	Bayinbuluke	Meat–fat	0.94	0.21	0.29
	n = 40	Duolang	Meat–fat	0.93	0.18	0.45
	n = 40	Cele Black	Lambskin	0.89	0.21	0.11
	n = 81	Kazakh	Meat–fat	0.69	0.31	0.41
	n = 38	Tan	Wool	0.97	0.12	0.28
	n = 30	AUW	Meat	0.53	0.55	0.42
	n = 188	Tibetian	Coarse wool	0.87	0.17	0.27
	n = 146	Yunnan	Meat–wool–lambskin	0.97	0.03	0.33
	n = 44	YSFW	Meat–wool–milk	0.42	0.45	0.22
	n = 86	Liangshan Wool	Wool–meat	0.70	0.31	0.15
	n = 30	Liangshan Black	Meat	0.95	0.02	0.17
Europe	n = 43	Poll Dorset	Meat	0.42	0.38	0.47
	n = 55	Romney	Meat	0.42	0	0.25
	n = 41	Dorper	Meat	0.49	0.29	0.28
	n = 62	Merino Horned	Wool–meat	0.50	0.10	0.13
	n = 50	Merino Polled	Wool–meat	0.45	0.14	0.2
	n = 55	EFD	Milk	0.31	0.15	0.08
	n = 38	Chinese Merino	Meat–wool	0.36	0	0.16
Africa	n = 72	Morocco	Meat	0.66	0.26	0.22
	n = 30	D’man	Meat	0.67	0.18	0.17
	n = 40	Coopworth	Meat–wool	0.49	0.24	0.16
	n = 31	Dairy Meade	Meat–milk	0.45	0.02	0.07

Note: EFD: East Friesian Dairy; LHT: Large-tail Han sheep; STH: Small-Tail Han sheep; AUW—Australian White; FLP—France local population.

**Table 4:** Associations of *MAST4* InDels with litter size in different parity groups in AUW ewes.

Loci	Parity/Average	Sample Sizes	Genotypes (Mean ± SE)			*p*-Values
			II	ID	DD	
P3-ins-29 bp	I	394	1.32 ± 0.05 (n = 68)	1.37 ± 0.03 (n = 229)	1.30 ± 0.04 (n = 97)	0.513
	II	311	1.31 ± 0.07 (n = 54)	1.40 ± 0.03 (n = 178)	1.51 ± 0.06 (n = 79)	**0.043**
	III	204	1.29 ± 0.07 (n = 35)	1.47 ± 0.04 (n = 119)	1.48 ±0.07 (n = 50)	0.063
	Average	425	1.36 ± 0.04 (n = 73)	1.39 ± 0.02 (n = 247)	1.38 ± 0.04 (n = 105)	0.788
P5-del-24 bp	I	266	1.39 ± 0.1 (n = 18)	1.30 ± 0.03 (n = 174)	1.31 ± 0.05 (n = 74)	0.769
	II	212	1.69 ± 0.2 (n = 13)	1.35 ± 0.04 (n = 137)	1.44 ± 0.07 (n = 62)	**0.031**
	III	138	1.75 ± 0.1 (n = 8)	1.49 ± 0.05 (n = 93)	1.32 ± 0.08 (n = 37)	**0.039**
	Average	221	1.71 ± 0.2 (n = 13)	1.39 ± 0.03 (n = 145)	1.40 ± 0.05 (n = 63)	**0.011**
P6-del-21-bp	I	179	1.32 ± 0.04 (n = 109)	1.46 ± 0.07 (n = 59)	1.55 ± 0.20 (n = 11)	0.112
	II	146	1.36 ± 0.05 (n = 87)	1.34 ± 0.06 (n = 50)	1.56 ± 0.33 (n = 9)	0.282
	III	95	1.43 ± 0.06 (n = 56)	1.41 ± 0.08 (n = 34)	2.00 ± 0.31 (n = 5)	0.052
	Average	186	1.37 ± 0.03 (n = 115)	1.39 ± 0.43 (n = 60)	1.64 ± 0.18 (n = 11)	**0.031**

Note: Bold values represent significance.

**Table 5:** *MAST4*-P5-del-24 bp genotypic distribution between mothers of single-lamb and multi-lamb in AUW ewes.

Parity	Types	Genotypes			χ^2^ Values	*df*	*p* Values
		II	ID	DD			
I	single	12	125	52	0.243	2	0.858
	multi	6	49	22			
II	single	6	90	37	46.903	2	**9.7428 × 10^−12^**
	multi	47	47	25			
III	single	2	48	26	6.828	2	**0.034**
	multi	6	45	11			

Note: χ^2^: chi-square value; the bold values mean significance.

## Data Availability

The data presented in this study are available in the article.
